# Vulcanizer Explosions

**Published:** 1880-11

**Authors:** 


### ARTICLE IV.

### *Vulcanizer Explosions.*

A Vulcanizer explosion occurred at a town in Pennsylvania, August 21st, through which a young man lost an arm. It is such a good example of the ignorance and foolhardiness sometimes displayed in the management of Vulcanizers, that we deem it worthy of a somewhat extended notice. From the account in a daily paper, it appears that the student had "taken his apparatus to Mr. Topper's blacksmith shop to assist him in getting up the proper temperature, and he got it up so well that the Vulcanizer exploded, a portion of it striking him in the arm near the elbow, inflicting a wound that necessitated amputation."

We have no further particulars of this deplorable affair than the above, but have heard it rumored that the vulcanizer was put into the blacksmith's forge, when the thermometer was known to be out of order.

Accompanying every Vulcanizer is a four-page circular, headed "PLEASE READ THESE DIRECTIONS CAREFULLY AND PRESERVE THEM FOR REFERENCE." We have abundant evidence that these circulars are seldom read by dentists; indeed, it is the common practice of some dentists to consign to the waste-basket all circulars that are sent them, even when their contents might be of vital importance in throwing light upon some imperfectly understood points. Now, if the preceptor of this young gentleman had preserved these Vulcanizer directions and posted them in his laboratory for the instruction of his student, this deplorable accident would probably never have occurred, as a thoughtful person would never think of placing a Vulcanizer in a forge, after giving these directions proper consideration. A proper heating apparatus is sent with every Vulcanizer, and the person who uses in its stead a cook stove, parlor stove, or blacksmith's forge, takes his life in his own hands.

As a precautionary measure, we copy a portion of the above-mentioned Vulcanizer directions, hoping that the re-publication may prevent carelessness in the use of Vulcanizers:

"This Vulcanizer has been tested by hydrostatic pressure, and has proved itself capable of withstanding five hundred pounds to the square inch, over six times the pressure at the vulcanizing point. This fact is given as an assurance of ample strength, but it forms no excuse for careless management.

If every dentist were thoroughly informed as to the nature and properties of steam, accidents from vulcanizers would be almost unheard of. For their information the following table is subjoined. The fact should be borne in mind that a vulcanizer is subject to the same laws and conditions as a steam boiler, which it is in fact; and although it is safe and easily operated, it may, by a little carelessness or ignorance in its management, become almost as dangerous as a bombshell.

### TABLE OF THE ELASTIC FORCE OF STEAM.

### FRENCH ACADEMY EXPERIMENTS.

| *Degrees of Temperature, Fahrenheit.* | *Elastic Force, in lbs. per square inch.* |
|---|---|
| 212. | 14.7 |
| 233.96 | 22.05 |
| 250.52 | 29.4 |
| 263.84 | 36.7 |
| 275.18 | 44.15 |
| 285.08 | 51.45 |

TABLE CONTINUED.—

| *Degrees of Temperature, Fahrenheit.* | *Elastic Force, in lbs. per square inch.* |
|---|---|
| 293.72 | 58.8 |
| 300.28 | 66.12 |
| 307.05 | 73.5 |
| 314.24 | 80.85 |
| 320.36 | 88.2 |
| 326.26 | 95.55 |
| 331.70 | 102.9 |
| 336.86 | 110.85 |
| 341.78 | 117.6 |
| 350.78 | 132.3 |
| 358.88 | 147. |
| 366.85 | 161.7 |
| 374.00 | 176.4 |
| 380 66 | 191.1 |
| 386.94 | 205.8 |
| 392.46 | 220.5 |
| 398.48 | 235.2 |
| 403.82 | 249.0 |
| 408.92 | 264.6 |
| 413.78 | 297.3 |
| 418.46 | 294. |

It will be noticed that as the temperature rises, the pressure of steam increases in a constantly increasing ratio for equal increments of heat; the pressure being more than doubled by the addition of fifty degrees to the temperature. This fact will show the *absolute necessity* of care and watchfulness while vulcanizing. For additional security, each vulcanizer is provided with a safety disk, which is intended to give way at about 340°. It must not be relied upon too implicitly, as no such arrangement can be made *absolutely certain* in its action. Moreover, as there is a constant liability in any kind of apparatus to get out of order, too much dependence should not be placed upon the indications of the thermometer. If the usual size flame does not heat the vulcanizer in about the usual time, the condition of the thermometer should be ascertained at once. The bulb will probably be found cracked or broken. A second tube and scale should always be kept on hand, ready to be substituted in case of any accident. If the thermometer case should be unscrewed from the vulcanizer top, a small cup will be seen in the nipple from which the case is removed. This cup should contain sufficient mercury to insure its touching the bulb of the tube when the thermometer case is screwed down properly. This makes a *metalic connection* between the thermometer bulb and vulcanizer cap, and is *absolutely necessary* for the proper indication of heat by the thermometer. If the safety plug should blow out at a degree of heat lower than 340°, it is probably because there is not sufficient mercury in the mercury cup and the thermometer should be removed and more mercury added.

In heating up, let on just flame enough to cover the bottom of the boiler, *not to flow up the sides.* A large flame overheats the outside, burns the packing, and damages the whole apparatus, without materially hastening the rise of the mercury.

If alcohol or kerosene is used, let the wick of the lamp be quite short. If gas is used, a flow of 2 to 2½ feet per hour is all that is required in heating up. After the mercury is up to the desired point, a very small flame will keep it there."

A great deal of unnecessary annoyance is experienced by dentists in the matter of blowing out the safety-disks, from the practice of filling or nearly filling the Vulcanizer with water. At least half an inch, or better, one inch of steam room should always be left. Water expands one-twenty-second of its volume by an increase of temperature from the freezing to the boiling point, and its rate of expansion increases with its temperature, so that at 320° it will have expanded at least one-tenth of its original volume. As water possesses but little elasticity, this force is practically irresistible. A Vulcanizer built of steel an inch thick could not stand the strain it would be subjected to if filled so that it had only one-twentieth of its capacity for steam room, and heated to the vulcanizing point. Some years ago, when the fusible safety valve was in use, a Whitney Vulcanizer was returned to the manufacturers, which was stretched, or enlarged in its diameter nearly a quarter of an inch. A series of inquiries revealed the facts: 1st. That the enlargement was gradual, several vulcanizations having been accomplished before the whole amount of enlargement was attained. 2d. The Vulcanizer had not been overheated. 3d. The dentist always used soft water and *always filled his Vulcanizer brim full.* This fact accounted for all the trouble. When the copper disk is used, the Vulcanizers cannot be subjected to such a heavy strain as the one before mentioned, as the disk will give way by pressure, irrespective of the temperature.

By experiment it is found that a No. 2 Hayes Boiler, if filled with cold water, will blow out the copper disk at about 310°. Under similar circumstances, with the No. 3 Hayes Boiler it will give way at about 260°, there being a greater mass of water to expand in the case of the No. 3 boiler. With the Whitney Vulcanizer, which can be filled nearer the top than the Hayes Boiler, the disk will give way at a still lower temperature.

In view of the foregoing, the absurdity of the following letter will be apparent:

"The Vulcanizer you sent me is a total failure. On Saturday it blew off at safety-valve four times, when the thermometer indicated 320°. We tried two disks instead of one, and they blew through. On yesterday the Vulcanizer seemed to work better, and had been standing at 320° for twenty-three minutes, when off it went, blowing through the bottom, etc."

An inquiry directed to these parties elicited the reply that "at the time the Vulcanizer *was full of water.*"

Only those who repair Vulcanizers can have any knowledge of the amount of abuse to which they are subjected. The wonder is not that they explode, but that explosions are so infrequent. The safety-disk is often replaced by a piece of metal too thick to give way under any reasonable amount of pressure, and nothing but an explosion with a narrow escape with life will convince some dentists of the foolhardiness of thus tampering with the only safeguard against careless overheating, to avoid the occasional annoyance occasioned by the blowing out of a disk. It is nothing uncommon to see the bottom of a Vulcanizer bulged into almost a hemispherical shape, and the sides expanded a quarter of an inch, the safety-valve plugged up, and the whole apparatus in such a condition as to betoken the most extreme neglect. In conclusion, we can only say that a Vulcanizer is not a thing to be trifled with, and that the person in charge of one should be careful, and well informed as to its proper use.—*Dental Advertiser.*

